# Carvedilol prevents impairment of the counterregulatory response in recurrently hypoglycaemic diabetic rats

**DOI:** 10.1002/edm2.226

**Published:** 2021-02-06

**Authors:** Rawad Farhat, Eliane de Santana‐Van Vliet, Gong Su, Levi Neely, Thea Benally, Owen Chan

**Affiliations:** ^1^ Department of Internal Medicine Division of Endocrinology, Metabolism and Diabetes University of Utah Salt Lake City UT USA; ^2^ Department of Cardiovascular Medicine Shanghai Wusong Central Hospital Shanghai China; ^3^ Department of Biology Utah Valley University Orem UT USA; ^4^ Department of Health, Exercise and Sports Sciences University of New Mexico Albuquerque NM USA

**Keywords:** adrenergic sensitivity, beta‐blocker, carvedilol, diabetes, hypoglycaemia, hypoglycaemia awareness, streptozotocin

## Abstract

**Aim:**

It has been suggested that repeated activation of the adrenergic system during antecedent episodes of hypoglycaemia contributes to the development of counterregulatory failure. We previously reported that treatment with carvedilol, a non‐specific β‐blocker, prevented the development of counterregulatory failure and improved hypoglycaemia awareness in recurrently hypoglycaemic non‐diabetic rats. The current study investigated whether carvedilol has similar benefits in diabetic rats.

**Methods:**

Recurrently hypoglycaemic streptozotocin‐diabetic rats (STZ+RH) were treated with carvedilol for one week prior to undergoing a hypoglycaemic clamp. Hypoglycaemia awareness was evaluated in streptozotocin‐diabetic rats made hypoglycaemia unaware using repeated injections of 2‐deoxyglucose.

**Results:**

Compared to hypoglycaemia‐naïve STZ‐diabetic controls, exogenous glucose requirements were more than doubled in the STZ+RH animals and this was associated with a 49% reduction in the epinephrine response to hypoglycaemia. Treating STZ+RH animals with carvedilol improved the epinephrine response to hypoglycaemia. Of note, neither recurrent hypoglycaemia nor carvedilol treatment affected the glucagon response in diabetic animals. Additionally, carvedilol treatment improved the feeding response to insulin‐induced hypoglycaemia in diabetic animals made ‘hypoglycaemia unaware’ using repeated injections of 2‐deoxyglucose, suggesting the treatment improved awareness of hypoglycaemia as well.

**Conclusion:**

Our data suggest that carvedilol may be useful in preventing impairments of the sympathoadrenal response and the development of hypoglycaemia unawareness during recurring episodes of hypoglycaemia in diabetic animals.

## Graphical abstract

Patients with type 1 diabetes lose the ability to recognize when their blood glucose levels go low, and this is due in part to loss of the sympathoadrenal response. Treatment of diabetic rats with low doses of carvedilol improves the sympathoadrenal response to hypoglycemia and their ability to recognize hypoglycemia.

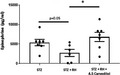

## 1 INTRODUCTION

Iatrogenic hypoglycaemia remains the most serious acute complication for patients with type 1 diabetes (T1D). While patients with T1D on intensive insulin therapy are at reduced risk for developing diabetic complications, the drawback is they experience a greater incidence of hypoglycaemia.^1^ This is due in part to an attenuated sympathoadrenal response to hypoglycaemia and the resulting loss of hypoglycaemia awareness, a syndrome known clinically as hypoglycaemia‐associated autonomic failure (HAAF).^2^, ^3^

As patients with T1D lose the ability to secrete glucagon within the first few years after diabetes onset, the sympathoadrenal response becomes crucial for the physiological recovery from hypoglycaemia. But, more importantly, the sympathoadrenal response is important for triggering symptomatic awareness of hypoglycaemia. Unfortunately, prior antecedent exposure to hypoglycaemia dramatically reduces this response and awareness of hypoglycaemia. While numerous mechanisms have been proposed to explain the pathophysiology of HAAF, we and others have shown that repeated activation of the adrenergic system may also contribute to the development of HAAF.^4^, ^5^, ^6^ Both repeated administration of epinephrine to healthy human subjects in the absence of hypoglycaemia and the administration of beta‐adrenergic blockers to type 1 diabetic patients during antecedent episodes of hypoglycaemia have supported this notion.^7^, ^8^

While the activation of adrenergic receptors in the ventromedial hypothalamus (VMH) appears to be important for augmenting the epinephrine response to an *acute* bout of hypoglycaemia,^9^, ^10^, ^11^ repeated activation of VMH adrenergic receptors during antecedent episodes of hypoglycaemia may in fact be detrimental to the counterregulatory response.^4^, ^5^ Importantly, Beverly and colleagues previously showed that release of norepinephrine in the VMH was not impacted by antecedent hypoglycaemia, despite loss of the sympathoadrenal response.^12^, ^13^ Taken together, the data suggest that moderate activation of the sympathoadrenal system may be beneficial for the recovery from hypoglycaemia,however, repeated robust activation of the adrenergic system may lead to the development of HAAF as reported by Yimagou and colleagues.^8^ This suggests that mild adrenergic blockade may be suitable for preventing the development of HAAF. We previously demonstrated that low doses of the non‐specific beta‐adrenergic blocker, carvedilol, was not only effective at improving the sympathoadrenal response in non‐diabetic rats subjected to recurring episodes of hypoglycaemia, but it also improved hypoglycaemia awareness in animals with impaired awareness of hypoglycaemia.^4^ Hence, in the current study, we examined whether this treatment can be used to improve hypoglycaemia awareness and the sympathoadrenal response in recurrently hypoglycaemic diabetic rats, a more clinically relevant model.

## 2 METHODS

Adult male Sprague Dawley rats (CD:SD, strain 001; Charles River, Wilmington, MA, USA) of 7–8 weeks of age and weighing ~300 g were individually housed in conventional rat cages at the University of Utah's Comparative Medicine Center in temperature‐ (22 ± 2°C) and humidity‐controlled rooms. Cages were lined with wood chip bedding, and the animals were provided with environmental enrichment in the form of a red acrylic tube and a gnawing block. The animals had free access to rodent chow (Envigo Teklad; Madison, WI, USA) and water and were conditioned to a 12 hours light/dark cycle (lights on between 07:00 hours and 19:00 hours) for 1 week before experimental manipulation. The principles of laboratory animal care were followed, and experimental protocols were approved by the Institutional Animal Care and Use Committee at the University of Utah.

### 2.1 *Study 1*: *Evaluating the Effects of Carvedilol Treatment on the Counterregulatory Hormone Responses to Hypoglycaemia*

#### 2.1.1 Streptozotocin (STZ)‐induced diabetic animal models

All animals were made diabetic using a single intraperitoneal injection of streptozotocin (STZ) dissolved in saline (60 mg/kg; Sigma‐Aldrich, St. Louis, MO, USA). A 10% sucrose solution was provided in place of the drinking water for the first 24 hours after STZ administration to prevent hypoglycaemia. After that, the animals received regular drinking water. Diabetes was maintained for a period of 2 weeks, and during this time, blood glucose was monitored twice daily with the AlphaTRAK2 glucometer (Abbott Laboratories; Chicago, IL, USA), and a variable subcutaneous dose of protamine zinc insulin (Boehringer Ingelheim; Duluth, GA, USA) was administered once per day to keep morning glucose levels below 500 mg/dL.

#### 2.1.2 Surgery:

**FIGURE 1:**
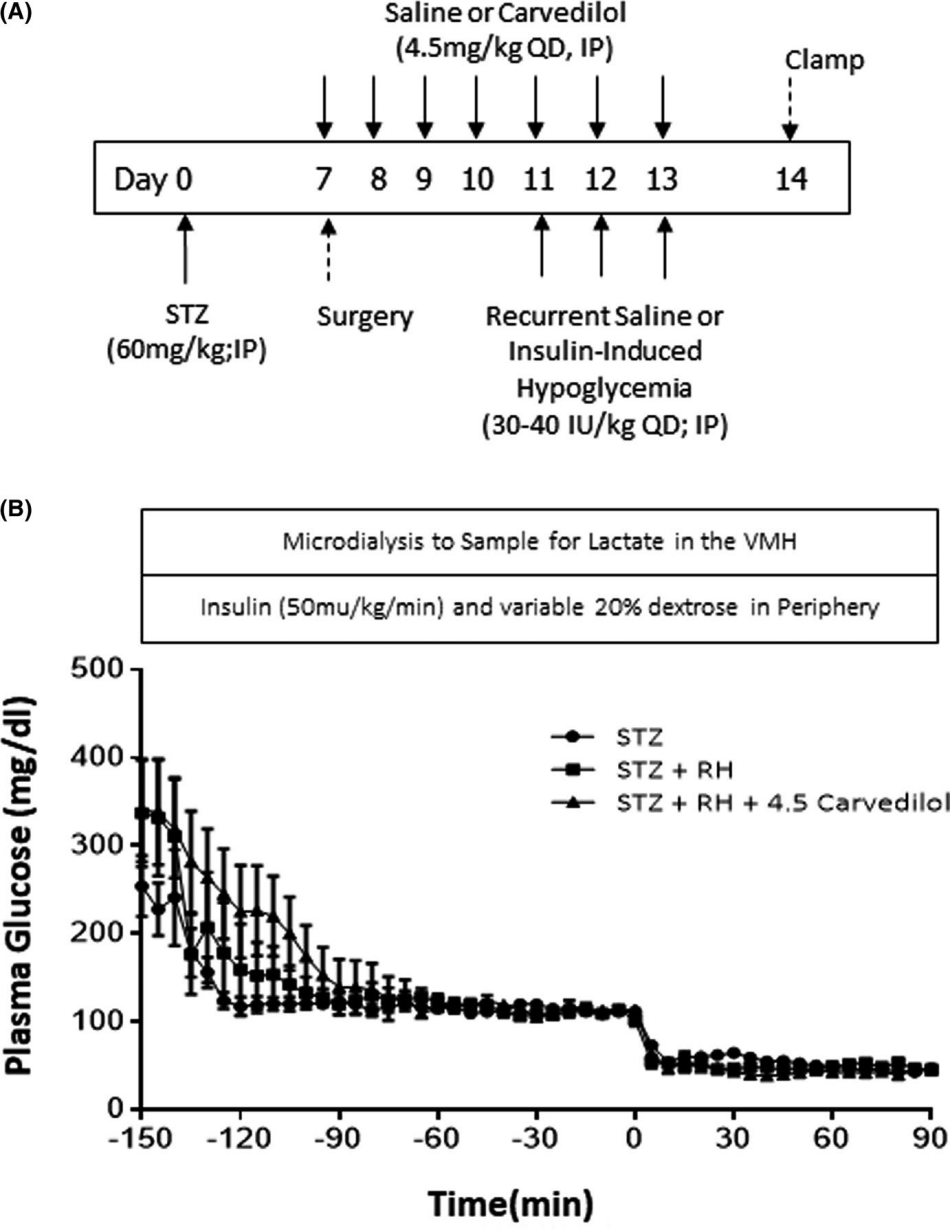
A, *Hypoglycaemic Clamp Study Design*. Schematic outlining the treatment time course. Diabetes was induced on day 0. Surgery for the implantation of catheters and microdialysis guide cannulas was performed one week later. Saline or carvedilol treatments were administered daily starting on day 7 and continued until day 13. Recurrent saline or insulin treatments was conducted from day 11–13. The hypoglycaemic clamp was conducted on Day 14. B, *Plasma glucose concentrations throughout the clamp procedure*. STZ Controls are represented as circles. STZ +recurrently hypoglycaemic diabetic (RH) animals are shown as squares, the RH diabetic rats treated with 4.5 mg/kg carvedilol (STZ+RH+4.5 Carvedilol) are shown as solid triangles. The hypoglycaemic phase of the study was from 0’ to 90’

One week after STZ administration Figure 1A, the rats underwent surgery for the implantation of vascular catheters and microdialysis guide cannulas (Amuza Inc, San Diego, USA) as described previously.^14^ Three days after surgery, the animals were randomly segregated into one of three groups: (1) STZ +saline (STZ; n = 7), (2) STZ +recurrent hypoglycaemia (STZ+RH; n = 6) or (3) STZ +recurrent hypoglycaemia +4.5 mg/kg carvedilol (STZ +RH +4.5 Carvedilol; n = 7).

#### 2.1.3 Recurrent Hypoglycaemia

Recurrent hypoglycaemia was initiated 3 days before the terminal clamp procedure or isoproterenol test. Hypoglycaemia was induced using a single intraperitoneal injection of regular insulin (30–40 IU/kg, Humulin R, Eli Lilly, Indianapolis, IN, USA). Plasma glucose concentrations were monitored through a tail nick every 30 minutes using an AlphaTRAK 2 glucometer (Abbott Laboratories, Chicago, IL, USA), and glucose levels were kept between 30 and 40 mg/dL for at least 1.5 hours (Supplemental Table 1). During recurrent hypoglycaemia, animals had free access to water, but not food. Following each episode of hypoglycaemia, plasma glucose levels were recovered to euglycemic levels by providing free access to food and water. In some cases, 0.5–1 mL of 20% dextrose was injected intraperitoneally to aid recovery if the animals did not eat on their own. Once recovered, the animals were returned to their home cages. This procedure was repeated once daily for 3 consecutive days. Control animals received a saline injection and underwent the same monitoring procedures.

#### 2.1.4 Carvedilol treatment

Carvedilol treatment was initiated ~3–7 days after the induction of diabetes. At 0800 h each day, the animals were administered a single intraperitoneal injection of carvedilol (4.5 mg/kg; Sigma‐Aldrich, St. Louis, MO, USA), which was dissolved in saline. STZ and STZ+RH animals received a daily saline injection in place of carvedilol. Carvedilol treatment lasted for a total of one week and continued throughout the 3 days of recurrent saline or insulin‐induced hypoglycaemia treatment Figure 1A. On each of the three days that the animals underwent the recurrent saline or recurrent hypoglycaemia treatment procedure, the saline or insulin injection was administered one hour after carvedilol was given. The beta‐blocker was not given on the day of the clamp studies.

#### 2.1.5 Stepped hyperinsulinemic‐euglycemic‐hypoglycaemic clamp

The day after the last bout of hypoglycaemia, following an overnight fast, bilateral 1 mm microdialysis/microinjection probes (Azuma Inc; San Diego, CA, USA) were inserted through the guide cannulas of the animals into the VMH and their venous catheter was connected to the infusion pumps. The microdialysis probes were constantly perfused with artificial extracellular fluid (aECF) at a rate of 1.5 µl/min.^15^ After a 2.5 hour recovery period, baseline blood samples were collected, and subsequently, microdialysate samples were collected every 10 minutes over the course of 30 minutes to evaluate baseline lactate concentrations. Once baseline microdialysate samples were collected, a constant insulin (50 mU/kg/min) and a variable 35% glucose infusion were started to lower and maintain plasma glucose levels at 110+/−10 mg/dL for the euglycemic phase of the study and subsequently, at 50+/−5 mg/dL for the hypoglycaemic phase of the study Figure 1B. Blood samples were collected every 5 minutes to assess plasma glucose concentrations using an Analox GM9 glucose analyser (Analox Instruments, Stourbridge, UK). Plasma samples were collected at baseline (−185′) and again at 0 (end of euglycemia), 30, 60 and 90 minutes during the hypoglycaemic clamping phase to measure the counterregulatory hormone responses to hypoglycaemia. Once the plasma was collected and frozen at −20°C (or −80°C for the catecholamine samples), the erythrocytes were re‐suspended in an equivalent volume of artificial plasma and re‐infused back into the animal to prevent volume depletion and anaemia. The primary end‐point of the hypoglycaemic clamp study was to determine whether carvedilol improved the counterregulatory hormone response in recurrently hypoglycaemic diabetic animals. At the end of the experiment, the animals were euthanized with an intravenous injection of sodium pentobarbital and the brains were rapidly removed, frozen and stored at −80°C until they were analysed. Accuracy of probe position was histologically verified and only those animals with properly positioned probes were included in the analysis.

*Hormone and Microdialysate Analysis*. Plasma catecholamine concentrations were analysed by ELISA (Abnova), while plasma glucagon concentrations were determined using commercially available radioimmunoassay kits (Millipore). Microdialysate lactate concentrations were analysed using a fluorometric assay (Biovision Inc.).

### 2.2 Study 2: Evaluating the effects of carvedilol treatment on adrenergic sensitivity

**FIGURE 2:**
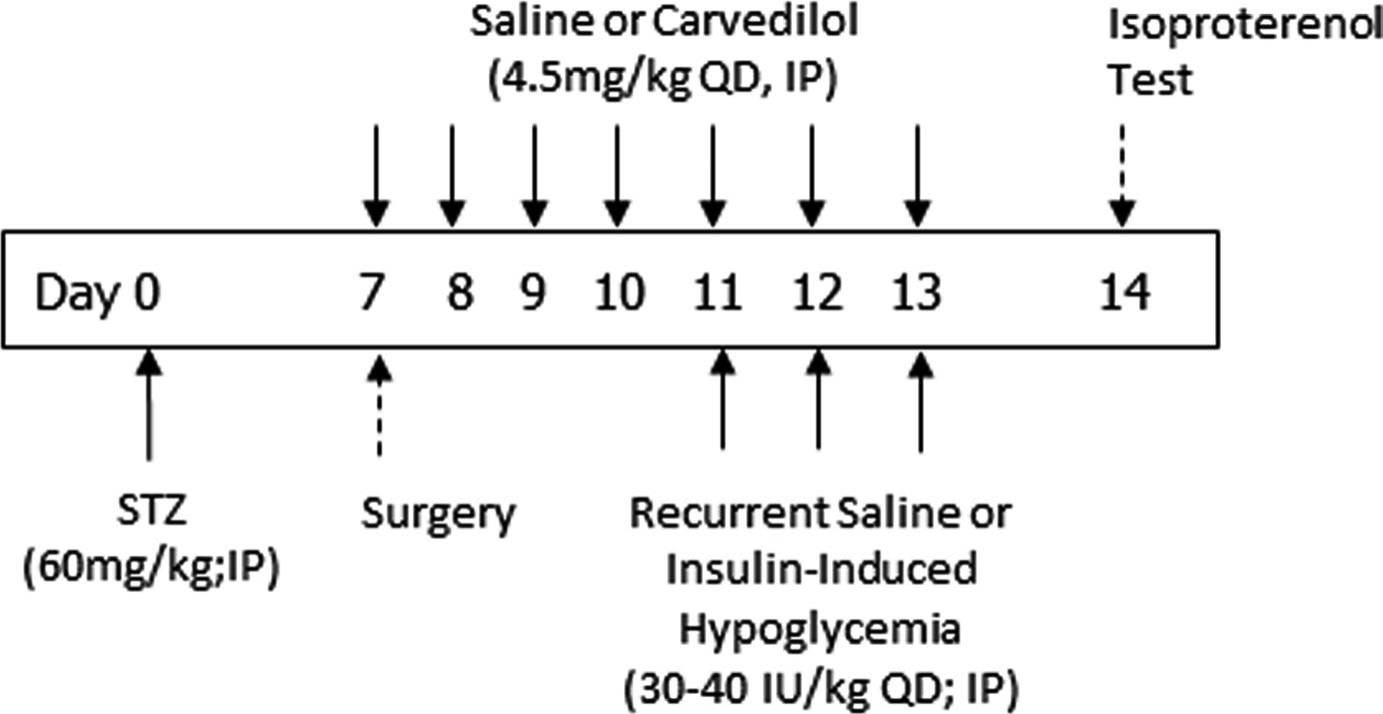
*Isoproterenol Test*. Schematic outlining the treatment time course. Diabetes was induced on day 0. Saline or 4.5 mg/kg carvedilol treatment was initiated on day 7 and continued until day 13. Recurrent saline or insulin treatments were given from days 11–13. The isoproterenol test was conducted on day 14 in lightly anesthetized rats

To establish whether recurrent hypoglycaemia or carvedilol treatment altered adrenergic sensitivity, we assessed adrenergic sensitivity in our diabetic animals using an isoproterenol test where a low dose of the non‐specific β‐adrenergic receptor agonist, isoproterenol (50 µg/kg, IV; Sigma‐Aldrich, St Louis, MO, USA), was administered and the chronotropic effect on heart rate was assessed as a marker of adrenergic sensitivity. One week after STZ administration, all of the animals underwent surgery to have a single vascular catheter inserted into the right jugular vein and started either daily saline or daily carvedilol treatment Figure 2. Four days after surgery, the animals started their respective recurrent treatment protocols for three consecutive days, receiving either saline or insulin injections, as described above. The day after the final treatment, the animals were lightly anesthetized with 0.5% isoflurane, laid supine, shaved and connected to an electrocardiogram (ECG) using three alligator leads. This dose of isoflurane was insufficient to prevent a toe‐pinch response and did not affect baseline heart rate.^16^, ^17^ Once stable, basal heart rate was recorded for a period of 10 minutes before isoproterenol (10 µg/kg,Sigma‐Aldrich, St. Louis, MO) was administered through the venous catheter to the following groups of animals (n = 4 per group): (1) STZ, (2) STZ+RH and (3) STZ+RH+4.5 mg/kg carvedilol. The change in heart rate was then monitored over the course of 10 minutes and analysed using the ADInstruments LabChart Pro 7.

### 2.3 Study 3: Evaluating the effects of carvedilol treatment on hypoglycaemia awareness

**FIGURE 3:**
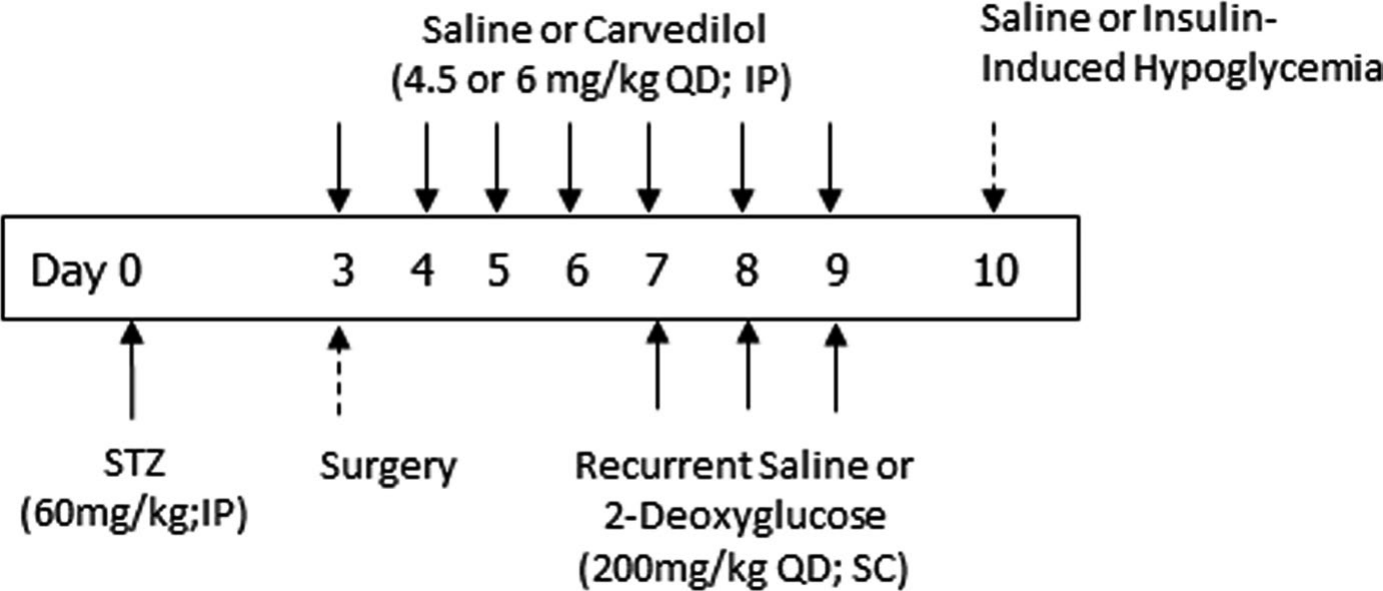
*Evaluating Hypoglycaemia Awareness*. Schematic outlining the treatment time course. Diabetes was induced on day 0. All rats had catheters implanted three days after induction of diabetes. Saline or carvedilol (4.5 or 6 mg/kg) treatments were initiated on day 3 and continued until day 9. Recurrent saline or 2‐deoxyglucose treatments were given once daily from days 7–9. On day 10, the animals were given either subcutaneous injections of saline or insulin, and food intake was assessed to evaluate hypoglycaemia awareness

To evaluate whether carvedilol improved awareness of hypoglycaemia, we treated rats that were made ‘impaired aware of hypoglycaemia (IAH)’ using three single daily subcutaneous injections of 2‐deoxyglucose (2DG, 200 mg/kg; Sigma‐Aldrich, St. Louis, MO, USA), and we subsequently measured food intake in response to insulin‐induced hypoglycaemia as a surrogate marker of hypoglycaemia awareness Figure 3.^4^ 2DG was administered for 3 consecutive days to mimic the three days of recurrent hypoglycaemia described above. Carvedilol treatment lasted for one week and on the days that 2DG was given, carvedilol treatment was administered one hour prior to the 2DG injection. One day after the last dose of 2DG was administered, non‐fasted diabetic rats were randomly assigned to one of the following treatment groups (recurrent treatment +final treatment): (1) saline +saline (n = 6), (2) saline +insulin (n = 6), (3) 2DG + insulin (n = 7), (4) 4.5 mg/kg carvedilol and 2DG + insulin (n = 9) or (5) 6 mg/kg carvedilol and 2DG + insulin (n = 6). For the final treatment to evaluate hypoglycaemia awareness, the rats were given either a single subcutaneous injection of saline or a combination of regular and NPH insulin (475 U/kg and 400 U/kg, respectively) to induce hypoglycaemia. This dose of insulin was necessary to induce and maintain a consistent hypoglycaemic stimulus in all the diabetic animals for the entire period that food consumption was evaluated,lower doses of insulin were insufficient to maintain glucose levels at ~50 mg/dL once the animals started to eat. The dose of insulin used was comparable to the total amount of insulin that was required to maintain hypoglycaemia using glucose clamp procedures while food consumption was evaluated (data not shown). Tail vein glucose and food consumption were evaluated every 2 hours and total food intake over the course of 4 hours was used as a surrogate measure of hypoglycaemia awareness. The primary end‐point of the awareness study was to determine whether carvedilol prevented the development of IAH (ie whether it increased food intake in response to insulin‐induced hypoglycaemia).

### 2.4 Statistical analysis

Treatment effects were analysed using one‐ (for AUC GIR, lactate, glucagon, epinephrine and food consumption data) or two‐way (for plasma glucose data) analysis of variance (ANOVA) for independent or repeated measures as appropriate, followed by Tukey's pairwise comparisons using Prism GraphPad™ 9.0 statistical software. *p* ≤ 0.05 was set as the criterion for statistical significance. Sample size required for all studies was determined using statistical power calculations based on our prior experience with these experiments, with a *p* < 0.10 for the beta error and *p* < 0.05 for the alpha error.

## 3 RESULTS

### 3.1 Plasma glucose and insulin concentrations and glucose infusion rates during the glucose clamp

**FIGURE 4:**
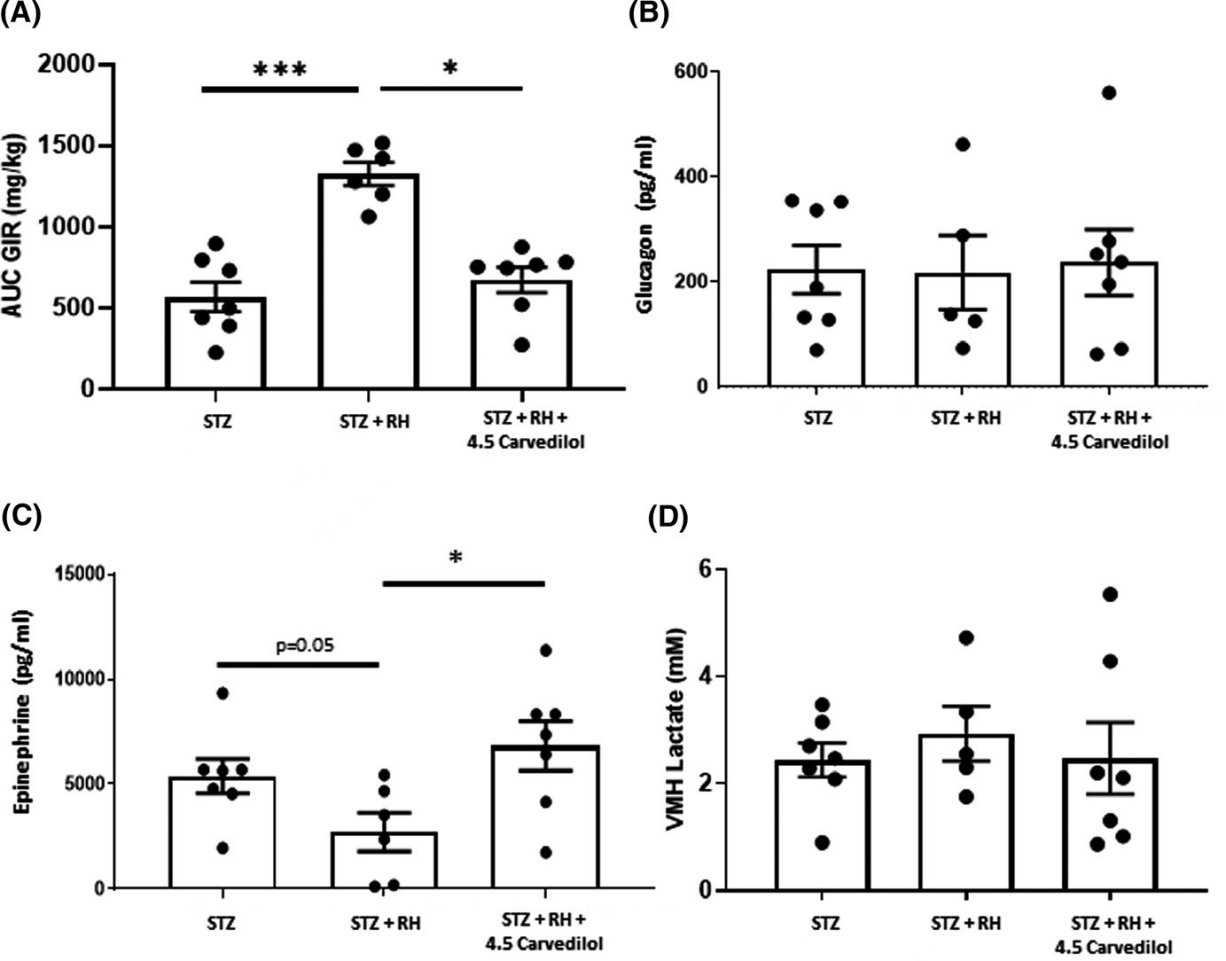
A, Total glucose infused over the final 30 min of the hypoglycaemic clamp procedure. B, Peak plasma glucagon and C, peak epinephrine responses during the hypoglycaemic phase of the clamp; and D, baseline extracellular lactate concentrations in the ventromedial hypothalamus of diabetic (STZ), recurrently hypoglycaemic diabetic (STZ+RH) and recurrently hypoglycaemic diabetic animals treated with 4.5 mg/kg carvedilol (STZ+RH+4.5 carvedilol). Data presented as mean ± SEM. *p < 0.05; ***p < 0.001

Plasma glucose concentrations during the hyperinsulinemic‐euglycemic phase of the clamp were similar between all of the treatment groups and fell to similar levels during the hypoglycaemic phase Figure 1B. Plasma insulin concentrations were similar between the treatment groups throughout the study Table 1. Despite similar plasma glucose and insulin concentrations, exogenous glucose requirements were significantly higher in diabetic animals exposed to recurring episodes of hypoglycaemia Figure 4A. In contrast, treatment with carvedilol reduced exogenous glucose requirements to normal.

**TABLE 1:** Plasma insulin concentrations (uU/ml) at baseline and at the end of the hypoglycaemic clamping procedure

	Baseline	Hypoglycaemia
STZ (n = 7)	7 ± 4	4367 ± 363
STZ +RH (n = 6)	5 ± 2	4547 ± 316
STZ +RH +4.5 Carvedilol (n = 7)	8 ± 4	4321 ± 175

Note

No differences were observed between the treatment groups either at baseline or during the hypoglycaemic clamp. Data presented as mean ± SEM.

### 3.2 Counterregulatory hormones responses during the hypoglycaemic clamp

Peak plasma glucagon responses to hypoglycaemia were similar between all three treatment groups Figure 4B. In contrast, recurrent hypoglycaemia attenuated the epinephrine response to hypoglycaemia in STZ‐diabetic animals (*p* = 0.05 vs STZ, Figure 4C, whereas treatment with 4.5 mg/kg of carvedilol restored the epinephrine response in recurrently hypoglycaemic diabetic rats.

### 3.3 VMH lactate

Recurrent hypoglycaemia did not alter VMH lactate concentrations in STZ‐diabetic rats. Treatment with carvedilol also did not affect VMH lactate concentrations in diabetic rats Figure 4D.

### 3.4 Isoproterenol test

**FIGURE 5:**
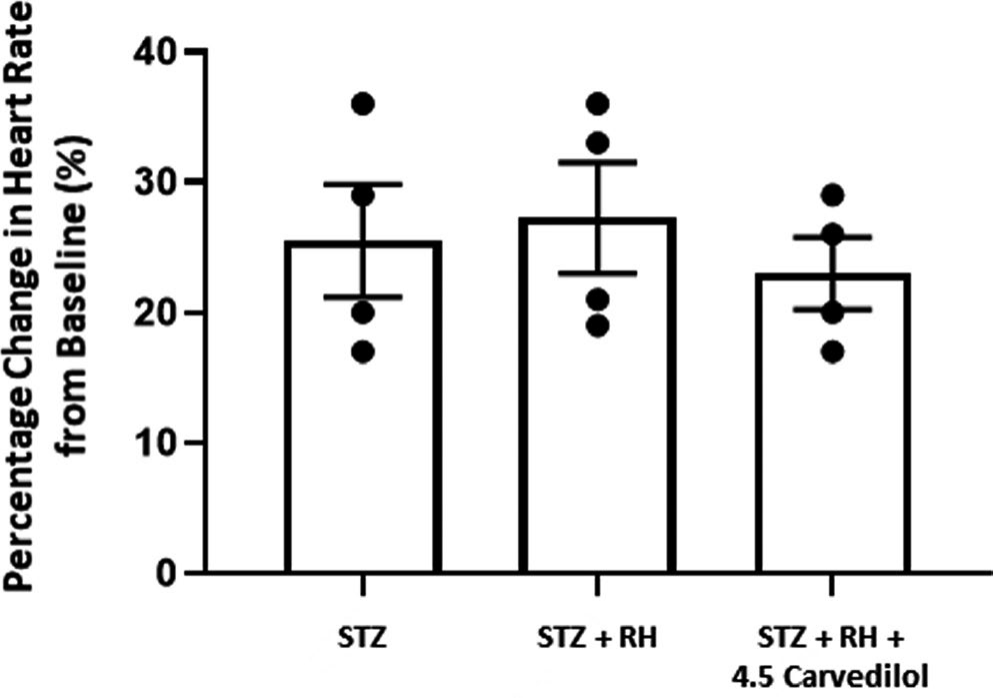
*Isoproterenol test*. This figure shows percentage change in heart rate from baseline levels in diabetic (STZ), recurrently hypoglycaemic diabetic (STZ+RH) and recurrently hypoglycaemic diabetic rats treated with 4.5 mg/kg carvedilol (STZ+RH+4.5 carvedilol) following intravenous administration of isoproterenol. There was no significant difference between the three treatment groups. Data presented as mean ±SEM.

All animals exhibited similar chronotropic responses to isoproterenol administration, suggesting recurring exposure to hypoglycaemia did not alter adrenergic sensitivity in diabetic rats and treatment with low doses of carvedilol had no significant effect on adrenergic sensitivity Figure 5.

**FIGURE 6:**
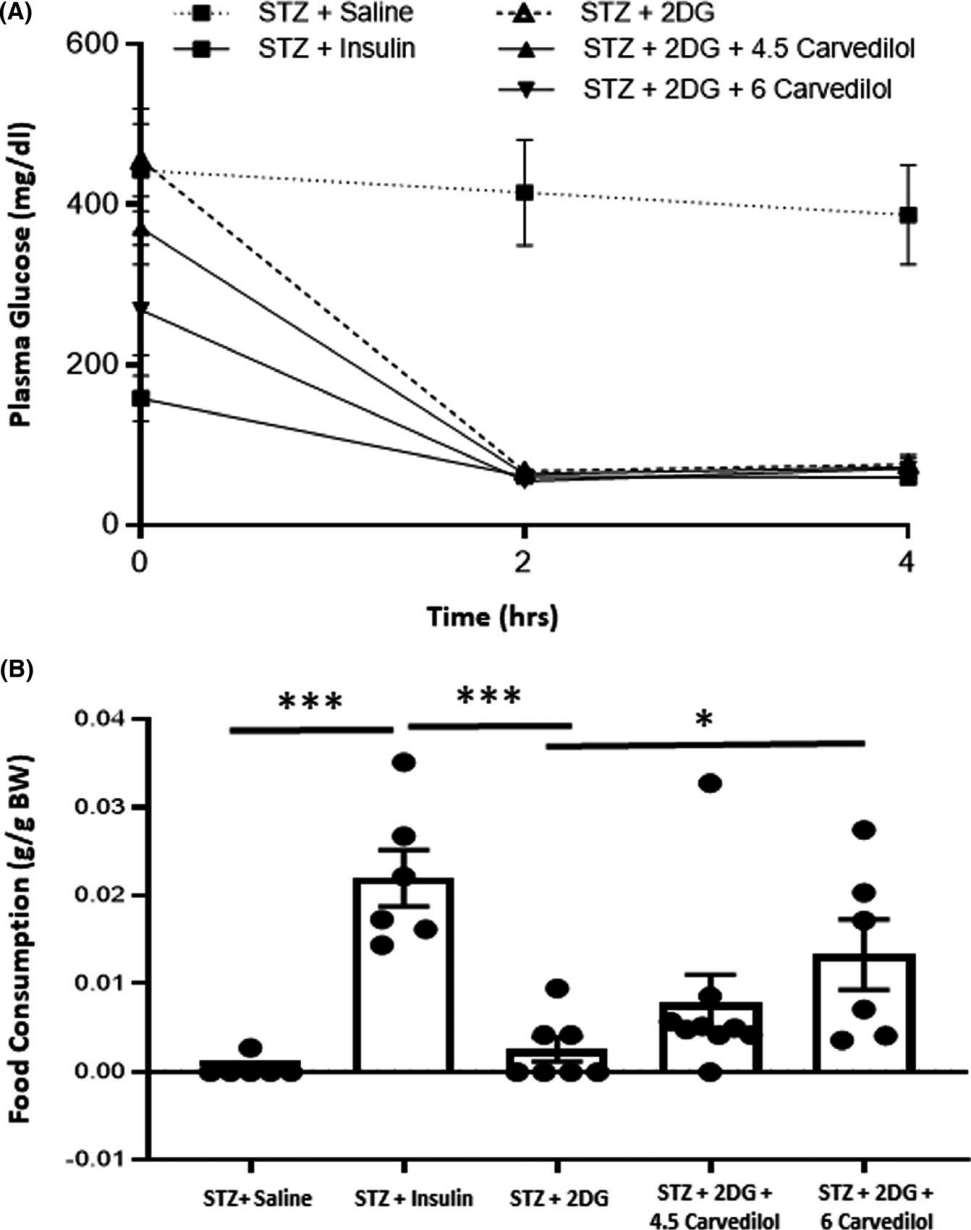
*Evaluation of hypoglycaemia awareness*. A, Blood glucose levels during the induction of hypoglycaemia. B, Diabetic rats made hypoglycaemic (STZ+Insulin) consumed significantly (***p < 0.001) more food than hypoglycaemia‐naïve rats given a saline injection (STZ+Saline). Diabetic rats treated with 2‐deoxyglucose (STZ+2DG) consumed significantly less food when hypoglycaemic compared to the STZ+Insulin group (***p < 0.001, 2DG+Insulin vs Saline+Insulin). Although treatment with 4.5 mg/kg carvedilol increased food consumption in STZ+2DG diabetic animals, the increase did not reach statistical significance. In contrast, when the STZ+2DG animals were treated with 6 mg/kg carvedilol, the animals consumed more food than the STZ+2DG group (*p < 0.05). Data presented as mean ± SEM

### 3.5 Carvedilol improved hypoglycaemia awareness in diabetic rats

Blood glucose concentrations in diabetic animals given a saline injection on the experiment day (STZ+Saline) remained at hyperglycaemic levels Figure 6A. During the induction of hypoglycaemia on the experiment day, plasma glucose concentrations during the hypoglycaemic phase were well matched between treatment groups. Compared to diabetic rats that were treated with saline on the day of the experiment, plasma glucose concentrations in diabetic rats that received insulin (STZ+Insulin) reached moderate hypoglycaemic levels and they consumed significantly more food in response to insulin‐induced hypoglycaemia Figure 6B. This suggests that hypoglycaemia or awareness of hypoglycaemia triggers a robust feeding response in STZ‐diabetic rats. On the other hand, treatment with 2DG (STZ+2DG) to induce IAH reduced food consumption in the diabetic animals. Although treatment with 4.5 mg/kg of carvedilol increased food consumption slightly in the 2DG‐treated diabetic animals, this rise did not reach statistical significance. We therefore evaluated a slightly higher dose of carvedilol, and treatment with 6 mg/kg of carvedilol significantly increased food intake in 2DG‐treated diabetic animals, suggesting improved awareness of hypoglycaemia with the higher dose of carvedilol.

## 4 DISCUSSION

Insulin remains the sole therapy for patients with type 1 diabetes (T1D), and despite advances in insulin development, iatrogenic hypoglycaemia remains a common problem for those patients seeking to reach optimal glycemic control. While intensive insulin treatment is effective at reducing the incidence of diabetic complications, the risk for hypoglycaemia increases dramatically. Additionally, exposure to hypoglycaemia reduces awareness of hypoglycaemia due to loss of the sympathoadrenal response.^18^ While the mechanisms underlying the development of HAAF are not clear, repeated activation of the adrenergic system may be one contributing factor.^5^, ^6^

Our previous studies in non‐diabetic rats demonstrated that treatment with low doses of the non‐specific β‐adrenergic receptor blocker, carvedilol, effectively prevented the development of counterregulatory failure and improved hypoglycaemia awareness in a rat model of HAAF.^4^ The current study reveals similar efficacy in a more clinically relevant model, a recurrently hypoglycaemic rat model of type 1 diabetes. We chose to investigate the potential of this third generation β‐blocker to prevent HAAF in recurrently hypoglycaemic diabetic rats because of its longstanding safety profile and its demonstrated benefits on glycemic management.^19^, ^20^, ^21^ Patients with type 2 diabetes who received carvedilol treatment reported improvements in metabolic parameters such as insulin sensitivity. But, more importantly, carvedilol did not adversely affect metabolic control in these patients. This is in contrast to what was observed with first and second generation β‐blockers which negatively impacted glycemic control.^22^ Currently, there are no clinical data available regarding the effects of carvedilol on metabolic control in type 1 diabetic patients.

It is interesting to note that recurrent hypoglycaemia did not affect the already attenuated glucagon response to hypoglycaemia in diabetic animals,^14^, ^23^, ^24^ and similarly, treatment with carvedilol also did not affect the glucagon response in recurrently hypoglycaemic diabetic rats. This result differs slightly from what we observed in recurrently hypoglycaemic non‐diabetic rats where recurrent hypoglycaemia attenuated the glucagon response and carvedilol treatment marginally improved the response, but not to the point of statistical significance. The regulation of glucagon secretion is complex, and different factors have been proposed to explain the blunted glucagon response to hypoglycaemia in type 1 diabetes, but the precise mechanism remains unclear. Most notable among these are changes in intraislet insulin that stems from loss of β‐cells and peripheral administration of insulin in patients with type 1 diabetes.^25^, ^26^, ^27^ Additionally, elevated somatostatin concentrations in type 1 diabetes have been shown to suppress glucagon secretion, with the administration of somatostatin antagonists showing promise in restoring glucagon secretion in animal models of type1 diabetes.^28^, ^29^, ^30^, ^31^, ^32^ The role of zinc co‐factors has also been proposed, but with some mixed results.^33^, ^34^ Lastly, we and others examined the contribution of central nervous system inputs to the alpha‐cells stemming from glutamatergic, GABAergic and noradrenergic neurotransmission as additional signals that may influence glucagon secretion, and while the release of these neurotransmitters may be affected by changes in local glucose and insulin concentrations in diabetes, a complete picture of their role is still outstanding.^10^, ^35^, ^36^, ^37^, ^38^

In contrast to the lack of effect on glucagon, the current study shows that recurring exposure to hypoglycaemia significantly attenuated the epinephrine response to a subsequent bout of hypoglycaemia. When low doses of carvedilol were administered to a recurrently hypoglycaemic rat model of type 1 diabetes, it effectively improved the sympathoadrenal response to hypoglycaemia as evidenced by a full restoration of the epinephrine response. This improved epinephrine response was accompanied by a reduction in exogenous glucose requirements during the hypoglycaemic phase of the clamp. These data support our earlier notion that low doses of carvedilol are effective in preventing impairments in the counterregulatory responses. Hence, a mild tempering of adrenergic stimulation is likely sufficient to improve the sympathoadrenal response and complete adrenergic blockade is not necessary. Our findings are similar to those reported by Yimagou and colleagues who reported that HAAF was elicited in healthy human subjects who exhibited larger epinephrine responses to hypoglycaemia, whereas those with smaller epinephrine responses, retained hypoglycaemia awareness, again suggesting that more profound adrenergic activation during antecedent episodes of hypoglycaemia may contribute to the development of HAAF.^6^

Our results were also comparable to those reported by Hirsch and colleagues who exhibited greater sympathoadrenal responses in type 1 diabetic patients after they were treated with propranolol.^7^ While administration of propranolol did not cause hypoglycaemia unawareness in this group of patients, it lowered the threshold for onset of hypoglycaemic symptoms. More importantly, however, hypoglycaemia awareness was improved through increased perception of diaphoresis. Although it is not possible to assess diaphoresis in rodents as they do not perspire, improvements in the feeding response to insulin‐induced hypoglycaemia that was observed in carvedilol‐treated diabetic animals with impaired hypoglycaemia awareness suggest that carvedilol likely improved symptomatic awareness of hypoglycaemia. Moreover, Ramanathan and colleagues reported that in healthy human subjects, adrenergic blockade during day 1 hypoglycaemia prevented suppression of the sympathoadrenal response during day 2 hypoglycaemia. These results are similar to those reported by Yimagou and colleagues who conducted the converse study where healthy human subjects were administered intravenous epinephrine repeatedly in the absence of hypoglycaemia on day 1 and this recapitulated the HAAF phenotype the following day.^6^ Taken together, these data support the notion that repeated adrenergic activation during antecedent bouts of hypoglycaemia plays a fundamental role in suppressing the sympathoadrenal response to subsequent episodes of hypoglycaemia and that β‐adrenergic receptor blockers may be a useful therapeutic strategy to prevent the development of HAAF and improve hypoglycaemia awareness in patients with T1D. In contrast to these findings, de Galan and colleagues reported that prior administration of epinephrine to healthy human subjects did not affect autonomic or neuroglycopenic symptoms, nor did it impair the counterregulatory hormone responses.^39^ Differences in the responses to epinephrine infusion between the two studies may be attributed to the amplitude and/or duration of epinephrine exposure, as well as the length of time between the epinephrine infusion and the clamp procedure.

To better understand the mechanism(s) through which carvedilol improves the sympathoadrenal response and hypoglycaemia awareness in our recurrently hypoglycaemic rat model of type 1 diabetes, we measured VMH lactate concentrations as we previously reported that recurrent hypoglycaemia raises VMH lactate concentrations in non‐diabetic animals and carvedilol restored these levels to normal. Poorly controlled diabetic rats already have higher lactate concentrations in the VMH compared to non‐diabetic animals,^40^ and notably, recurring exposure to hypoglycaemia did not increase these levels any further. Carvedilol treatment also did not reduce extracellular concentrations in the diabetic animals as we previously observed in the non‐diabetic animals. We postulate that elevated brain glucose levels in diabetes likely play a dominant role in raising brain lactate levels.^41^ Our data suggest that carvedilol may improve hypoglycaemia awareness in diabetic animals through other mechanisms—either by augmenting sympathetic autonomic output and/or through improved adrenergic sensitivity.

Given brain lactate concentrations were not affected by carvedilol treatment, we conducted an isoproterenol test to investigate whether a reduction in adrenergic sensitivity in peripheral tissues contributed to counterregulatory failure following antecedent exposure to hypoglycaemia^42^, ^43^, ^44^ and whether carvedilol improved adrenergic sensitivity. It has been suggested that a reduction in beta‐adrenergic sensitivity may help identify those patients who are at increased risk for hypoglycaemia unawareness.^43^ Both diabetes and recurring hypoglycaemia have been reported to decrease adrenergic sensitivity,^45^, ^46^ whereas avoidance of hypoglycaemia can improve adrenergic sensitivity.^42,47^ In the current study, we did not observe any differences in the chronotropic response to isoproterenol in our recurrently hypoglycaemic rat model of type 1 diabetes suggesting that, at least in the earlier stages of diabetes being studied, recurring exposure to hypoglycaemia does not reduce adrenergic sensitivity. Given this is the case, our data suggest that in the complex setting of poorly controlled type 1 diabetes, partial adrenergic receptor blockade may cause a compensatory autonomic response during hypoglycaemia that enhances the sympathoadrenal response to hypoglycaemia and hypoglycaemia awareness. The precise mechanisms are still not clear and are currently under investigation.

We subsequently evaluated whether carvedilol improved hypoglycaemia awareness in type 1 diabetic rats using a previously described model.^4^ This model uses food intake as a surrogate marker of hypoglycaemia awareness with the premise being that if the animal is aware that it is hypoglycaemic, it would consume more food compared to one that is less aware. We used repeated injections of 2DG to impair awareness of hypoglycaemia in our diabetic animals. Our previous results showed that low doses of carvedilol improved hypoglycaemia awareness in non‐diabetic recurrently hypoglycaemic rats.^4^ Here, we showed that insulin‐induced hypoglycaemia triggers a robust feeding response in STZ‐diabetic rats and treatment with 2DG significantly reduced the feeding response, suggesting the animals have impaired awareness of hypoglycaemia. It is interesting to note that even though treatment with 4.5 mg/kg of carvedilol increased the feeding response to hypoglycaemia, the increase did not reach statistical significance. In contrast, when a higher dose of carvedilol was used, the feeding response was increased significantly, suggesting we improved hypoglycaemia awareness with the higher dose of carvedilol. We speculate that the need for a higher dose of carvedilol to improve hypoglycaemia awareness in the 2DG‐treated diabetic animals may be due to the more profound glucoprivic effect that occurs with 2DG treatment compared to recurrent insulin‐induced hypoglyczemia. In support of this idea, recurrent 2DG administration was shown to have a more potent suppressive effect on the epinephrine response to hypoglycaemia compared to recurrent insulin‐induced hypoglycaemia.^48^ Although 2DG completely abolished the epinephrine response, recurrent insulin‐induced hypoglycaemia generally attenuated the epinephrine response.

A retrospective analysis was recently conducted on hospitalized diabetic patients who were taking beta‐blockers, either carvedilol or the selective beta‐blockers, metoprolol or atenolol, to examine whether the use of beta‐adrenergic blockers was associated with increased incidence of hypoglycaemia.^49^ The study reported that diabetic patients who were not on basal insulin therapy had increased odds of hypoglycaemia and this ration was greater for the selective beta‐blockers compared to carvedilol. Moreover, the odds of mortality stemming from hypoglycaemia were greater for those patients taking either a selective beta‐blocker or not taking any beta‐blocker, but notably, was reduced in those patients using carvedilol. This study suggests vasodilatory beta‐blockers such as carvedilol may reduce the incidence of hypoglycaemia in diabetic patients who are not on basal insulin therapy. However, what was not clear from the study was the dose of adrenergic blockers that was administered to the patients, which may influence hypoglycaemia outcomes.

Taken together, our data suggest that low doses of carvedilol improved the sympathoadrenal response to hypoglycaemia and hypoglycaemia awareness in a recurrently hypoglycaemic rat model of type 1 diabetes. We therefore conclude that mild adrenergic blockade may be helpful for restoring the lost sympathoadrenal response to hypoglycaemia following antecedent exposure to hypoglycaemia in diabetic animals and for improving hypoglycaemia awareness in type 1 diabetes.

## CONFLICTS OF INTEREST

No potential conflicts of interest relevant to this article were reported.

## AUTHOR CONTRIBUTION

RF, ES, GS, LV and TB researched the data. RF wrote the manuscript. RF and OC contributed to protocol development and reviewed/edited the manuscript. All authors have reviewed and approved the manuscript.

## Supporting information

Table S1

## Data Availability

The data sets generated during and/or analysed during the current study are available from the corresponding author on reasonable request.
