# Supplementary material for: Carvedilol prevents impairment of the counterregulatory response in recurrently hypoglycaemic diabetic rats
Source: Endocrinol Diabetes Metab. 2021 Feb 6;4(2):e00226. doi: 10.1002/edm2.226 (PMC8029566; doi:10.1002/edm2.226)
Supplement: Supplementary file 1 — Table S1 [file EDM2-4-e00226-s001.pdf]

Supplemental Table 1: Average daily blood glucose concentrations (mg/dl) in STZ-diabetic rats during the recurrent saline (STZ) or recurrent insulin treatments (STZ+RH and STZ+RH+4.5 Carvedilol groups). Data presented as mean ± S.E.M.

	**Time (min)**	**STZ (n=7)**	**STZ + RH (n=6)**	**STZ + RH + 4.5 Carvedilol (n=7)**
**Day 1**	0	413.5 ± 27.1	483.3 ± 42.1	478.3 ± 42.4
	30	384.5 ± 48.0	245.5 ± 52.9	215.0 ± 54.4
	60	363.5 ± 40.4	94.5 ± 11.0	90.8 ± 4.1
	90	376.8 ± 15.6	53.8 ± 12.3	80.3 ± 13.9
	120	367.8 ± 17.7	36.3 ± 3.4	35.3 ± 7.4
	150	360.3 ± 29.8	37.0 ± 4.5	30.0 ± 3.4
	180	365.0 ± 30.0	34.5 ± 4.9	31.0 ± 0.4
**Day 2**	0	429.3 ± 35.1	462.3 ± 43.6	448.0 ± 42.5
	30	416.8 ± 35.5	173.8 ± 64.9	124.3 ± 29.8
	60	375.5 ± 49.3	82.5 ± 23.2	59.0 ± 15.6
	90	372.8 ± 50.9	63.8 ± 21.0	38.5 ± 10.7
	120	377.3 ± 40.6	43.3 ± 6.4	30.0 ± 2.9
	150	382.5 ± 30.3	30.3 ± 5.6	31.0 ± 2.5
	180	370.3 ± 26.5	32.0 ± 4.7	31.5 ± 4.3
**Day 3**	0	414.8 ± 31.3	486.5 ± 64.5	453.0 ± 47.1
	30	426.5 ± 38.9	213.0 ± 56.2	213.5 ± 64.8
	60	382.0 ± 42.6	134.5 ±38.9	164.0 ± 73.6
	90	382.8 ± 26.4	75.0 ± 28.2	80.8 ± 27.4
	120	389.8 ± 36.1	46.3 ± 16.0	38.0 ± 4.4
	150	375.0 ± 41.7	31.5 ± 8.6	32.8 ± 4.9
	180	381.8 ± 43.8	37.8 ± 4.5	29.8 ± 3.4
